# The contribution of ultrasonographic findings to the prognosis of subacute thyroiditis

**DOI:** 10.20945/2359-3997000000253

**Published:** 2020-06-05

**Authors:** Muhammed Erkam Sencar, Murat Calapkulu, Davut Sakiz, Pinar Akhanli, Sema Hepsen, Hakan Duger, Ilknur Ozturk Unsal, Muhammed Kizilgul, Erman Cakal

**Affiliations:** 1 Department of Endocrinology and Metabolism University of Health Sciences Diskapi Yildirim Beyazit Training and Research Hospital Ankara Turkey Department of Endocrinology and Metabolism, University of Health Sciences, Diskapi Yildirim Beyazit Training and Research Hospital, Ankara, Turkey

**Keywords:** Subacute thyroiditis, ultrasonography, prognosis, permanent hypothyroidism

## Abstract

**Objective:**

Ultrasound assessment plays an important role in the diagnosis, and monitoring of subacute thyroiditis (SAT). However, the relationship between ultrasonographic findings and severity or prognosis of the disease is not known. The aim of the present study was to evaluate the relationship between bilateral and unilateral disease involvement and severity and prognosis of the disease.

**Subjects and methods:**

The initial laboratory values, ultrasonographic findings and long-term outcomes of 247 SAT patients were evaluated retrospectively.

**Results:**

In the ultrasonographic evaluation, bilateral involvement was detected in 154 patients, and unilateral involvement in 93 patients at the time of diagnosis. No significant difference was found between patients with bilateral or unilateral disease at the time of diagnosis in respect of the initial acute phase reactants. FT4 was significantly higher and TSH was significantly lower in the group with bilateral disease. Bilobar or unilobar disease on ultrasound at the time of diagnosis was not found to be a risk factor for permanent hypothyroidism or recurrence. The mean thyroid volume was determined to be 22.5 ± 10 cm^3^ at the beginning of treatment, and 11.2 ± 8 cm^3^ at the end of treatment. The initial thyroid volume and the thyroid volume at the end of treatment were significantly lower in patients who developed hypothyroidism.

**Conclusion:**

There was no relationship between initial acute phase reactants and bilateral or unilateral involvement of the disease. FT4 levels were found to be associated with the extension of the disease. The risk of recurrence and permanent hypothyroidism are not associated with the initial ultrasonographic aspect. Arch Endocrinol Metab. 2020;64(3):306-11

## INTRODUCTION

Subacute thyroiditis (SAT) is a self-limiting inflammatory thyroid disease with a history of viral etiology. SAT patients present with severe neck pain, a tender, stiff and enlarged thyroid gland and systemic symptoms such as fever and fatigue ( 1 - 3 ). Non-steroid anti-inflammatory drugs (NSAID) or steroid therapy is used for the treatment of SAT depending on the severity of the disease ( 4 ). While SAT patients are diagnosed by physical examination, clinical findings and laboratory tests, ultrasonography plays a major role in the differentiation of SAT from other painful thyroid diseases and Graves’ disease that it’s classic US findings are diffusely extended hypoechogenicity and diffusely incresed vascularization ( 5 ). Hypoechoic areas with blurred borders and reduced vascularization constitute typical ultrasonography findings of subacute thyroiditis, but the extension of these findings is uncertain for disease severity or prognosis. If the extension of ultrasonographic findings can be found to be associated with disease prognosis, ultrasonographic findings may have an impact on the choice of treatment beyond diagnosis. The aim of this study was to determine whether the detection of bilateral or unilateral disease on ultrasound makes a difference in SAT prognosis.

## SUBJECTS AND METHODS

### Study subjects, imaging technique, and interpretation

A retrospective analysis was made of 247 subjects who had diagnosed and treated in our center between 2014 and 2019. The study protocol was approved by the Ethics Committee of our center. The diagnosis of SAT was made with detection of elevated acute phase reactants and detection of classical ultrasonographic SAT findings in patients presenting with neck pain. In addition, all patients underwent thyroid function tests at the time of diagnosis and were used to support the diagnosis. Cytological confirmation was performed in patients with a suspected diagnosis ( 6 - 8 ). Subjects who run out from follow up and those missing in ultrasonographic or laboratory data were excluded from the study. All patients were treated with low dose methylprednisolone (≤ 16 mg). Patients with the hypothyroid phase of SAT ongoing for at least six months with a continuous need to levothyroxine treatment during this period were accepted as permanent hypothyroidism. Patients using levothyroxine before SAT diagnosis were excluded from the evaluation of permanent hypothyroidism.

Recurrence was admitted with the relapse of clinical signs, laboratory findings and ultrasonographic findings. Ultrasonographic examination was performed on all subjects at the time of diagnosis and at each control examination by authors experienced in ultrasonography (Hitachi HI Vision Prerius (Japan), with a linear 13MHz probe

. The volume of the thyroid was calculated using the ellipsoid volume formula (ml) (Length (cm) x Width (cm) x Thickness (cm) x π/6). Thyroid function tests and thyroid antibodies were measured with chemiluminescent immunoassay (Beckman Coulter, CA, USA). Normal ranges were defined as thyroid stimulating hormone (TSH): 0.38-5.33 mIU/L, fT4: 0.60-1.25 ng/dl, fT3: 2.28-4 ng/L, anti-thyroid peroxidase antibody (anti-TPO): 0-35 IU/mL, anti-thyroglobulin antibody (anti-TG): 0-40 IU/mL, C-reactive protein (CRP): 0-5 mg/L, erythrocyte sedimentation rate (ESR): 0-20 mL/hour, Leukocytes: 3570-11010 10^3^/uL, Neutrophil: 1690-7550 10^3^/uL.

### Statistical analysis

Statistical analyses were performed using SPSS software (Chicago, USA). Categorical data were expressed as frequencies and percentages (%). All continuous factors with normal distribution were shown as mean ± standard deviation (SD), and variables without normal distribution were shown as median (range) values. The Independent Samples t test was used for the analysis of continuous variables with normal distribution. The Mann-Whitney U test was used for the analysis of non-normally distributed variables. The associations between categorical variables were examined using Chi-square analysis. Binary multiple logistic regression test was used to evaluate the relationship between permanent hypothyroidism and variables (all factors displaying a significant p-value on the univariate tests). A value of *p <* 0.05 was admitted as statistically significant.

## RESULTS

The study included a total of 247 SAT patients, comprising 184 females and 63 males. The full demographic and baseline clinical data of the 247 patients are reported in Table 1 . The median follow-up time of patients was 29 (6.2-70) months. During ultrasonographic examination at diagnosis, bilobular disease involvement was detected in 154 (62%) patients and unilobular involvement in 93 (38%) patients. The mean thyroid volume was determined to be 22.5 ± 10 cm^3^at the beginning of treatment, and 11.2 ± 8 cm^3^at the end of treatment ( Table 1 ). There was no significant difference in terms of WBC, neutrophil count, ESR and CRP levels between patients with bilateral and unilateral disease at the time of diagnosis (p > 0.05). FT4 was significantly higher in the group with bilateral disease (p: 0.014), and TSH level was significantly lower in the group with bilateral disease (p: 0.018) ( Table 2 ).

**Table 1:** The full demographic and baseline clinical data of the 247 patients

	Total patients
n	247
Female/male (n)	184 (74.5%)/63 (25.5%)
Age (years)	44 ± 7.5
Follow up time (month)	29 (6.2-70)
Total Leukocytes (10^3^/uL)	9,262 ± 2,536
Neutrophils (10^3^/uL)	6,321 ± 2,123
ESR (mm/hour)	47.5 (21-120)
CRP (mg/L)	48(9-300)
TSH (mIU/L)	0.04 (0.001-15)
fT4 (ng/dL)	1.9 ± 1
fT3 (ng/dL)	5.1 ± 1.9
Anti-TPO positivity n (%)	7.2%
Anti-Tg positivity n (%)	6.8%
Bilateral disease involvement (n,%)	154 (62%)
Unilateral disease involvement (n,%)	93 (38%)
Initial thyroid volume (cm^3^)	22.5 ± 10
Last thyroid volume (cm^3^)	11.2 ± 8

Data are presented as mean ± SD, median (range); ESR: erythrocyte sedimentation rate; CRP: C-reactive protein; TSH: thyroid stimulant hormone; fT4: free thyroxine; fT3: free triiodothyronine; Anti-TPO: anti thyroid peroxidase antibody; Anti-TG: anti thyroglobulin antibody.

**Table 2:** Comparison of clinical data of patients with bilateral and unilateral disease

	Unilateral disease	Bilateral disease	p
n	93 (38%)	154 (62%)	
Female/male (n)	70 (75%)/23 (24%)	114 (74%)/40 (26%)	0.8
Age (years)	44 ± 7	44 ± 8	0.49
Total Leukocytes (10^3^/uL)	9,031 + 2,286	9,403 + 2,676	0.28
Neutrophils (10^3^/uL)	6,273 + 1,891	6,351 + 2,260	0.79
ESR (mm/hour)	45 (22-108)	48.5 (21-120)	0.27
CRP (mg/L)	48 (9-300)	54 (9-255)	0.88
TSH (mIU/L)	0.07 (0.0001-3.4)	0.03 (0.003-15.09)	0.018
fT4 (ng/dL)	1.69 + 0.79	2.08 + 1.1	0.014
fT3 (ng/dL)	4.4 ± 1.1	5.5 ± 2.2	0.016

Data are presented as mean ± SD, median (range); ESR: erythrocyte sedimentation rate; CRP: C-reactive protein; TSH: thyroid stimulant hormone; fT4: free thyroxine; fT3: free triiodothyronine; Anti-TPO: anti thyroid peroxidase antibody; Anti-TG: anti thyroglobulin antibody.

The recurrence rate was 12% in the current study. The age, gender, WBC, neutrophil count, ESR, CRP, TSH and fT4 levels were not significantly different in the recurrence group and the non-recurrence group (p > 0.05) ( Table 3 ). Detection of bilateral or unilateral disease involvement on ultrasound at the time of diagnosis was not found to be a risk factor for recurrence (p: 0.39). No relationship was determined between thyroid volume and recurrence rate (p: 0.65).

**Table 3:** Comparison of clinical data of recurrence and non-recurrence patients

	Non-recurrence group	Recurrence group	p
n	218 (88%)	29 (12%)	
Female/male (n)	159 (73%)/59 (27%)	25 (86%)/4 (14%)	0.12
Age (years)	44 ± 8	43 ± 6	0.24
Total leukocytes (10^3^/uL)	9,188 ± 2,492	9,758 ± 2,811	0.26
Neutrophils (10^3^/uL)	6,272 ± 2,036	6,643 ± 2,663	0.38
ESR (mm/hour)	46 (21-120)	54 (22-120)	0.95
CRP (mg/L)	48 (9-300)	55 (10-176)	0.89
TSH (mIU/L)	0.03 (0.001-15)	0.24 (0.03-4.3)	0.09
fT4 (ng/dL)	1.9 ± 1	1.4 ± 0.7	0.08
fT3 (ng/dL)	5.1 ± 2	4.7 ± 1.1	0.37
Anti-TPO positivity (%)	12.4%	8.3%	0.56
Anti-Tg positivity (%)	14.7%	9.1%	0.61
Bilateral/unilateral disease (n,%)	138 (63%)/80 (37%)	16 (55%)/13 (45%)	0.39
Initial Thyroid volume (cm^3^)	22.6 ± 10.2	21.7 ± 8.6	0.65

Data are presented as mean ± SD, median (range); ESR: erythrocyte sedimentation rate; CRP: C-reactive protein; TSH: thyroid stimulant hormone; fT4: free thyroxine; fT3: free triiodothyronine; Anti-TPO: anti thyroid peroxidase antibody; Anti-TG: anti thyroglobulin antibody.

The permanent hypothyroidism rate was 10% in the current study. The age, gender, WBC, neutrophil count, ESR, CRP, thyroid function tests were similar in the groups that developed permanent hypothyroidism and that did not develop it (p > 0.05) ( Table 4 ). The rate of permanent hypothyroidism was found to be significantly higher in anti-TPO and anti-Tg positive patients (p: 0.02 and p: 0.027 respectively). Bilateral or unilateral disease involvement on ultrasound at the time of diagnosis was not found to be a risk factor for permanent hypothyroidism (p: 0.38). The initial thyroid volume and the thyroid volume at the end of treatment were significantly lower in patients who developed hypothyroidism than in those without hypothyroidism (p: 0.02 and p: 0.03 respectively). Thyroid volume was not detected as an independent risk factor for developing permanent hypothyroidism (p: 0.49, r^2^: 0.168).

**Table 4:** Comparison of clinical data of patients with permanent hypothyroidism and without permanent hypothyroidism

	Patients without permanent hypothyroidism	Patients with permanent hypothyroidism	p
n	220 (90%)	24 (10%)	
Female/male (n)	161 (73%)/59 (27%)	20 (83%)/4 (17%)	0.28
Age (years)	44 ± 8	45 ± 7	0.64
Total leukocytes (10^3^/uL)	9,352 ± 2574	8,330 ± 1,847	0.08
Neutrophils (10^3^/uL)	6,398 ± 2170	5,608±1,471	0.11
ESR (mm/hour)	47.5 (21-120)	52 (24-120)	0.76
CRP (mg/L)	49 (9-300)	49 (9-191)	0.96
TSH (mIU/L)	0.035 (0.003-15)	0.04 (0.001-6.3)	0.60
fT4 (ng/dL)	1.9 ± 1	1.8 ± 0.9	0.70
fT3 (ng/dL)	5.1 ± 1.9	4.7 ± 2.3	0.50
Anti-TPO positivity (%)	9.7%	33.3%	0.02
Anti-Tg positivity (%)	11%	36.4%	0.04
Bilateral/unilateral disease (n,%)	141 (64%)/79 (36%)	13 (54%)/11 (46%)	0.33
Initial thyroid volume (cm^3^)	23 ± 10.1	18 ± 8	0.02
Last thyroid volume (cm^3^)	12 ± 8.3	7.2 ± 4.1	0.03

Data are presented as mean ± SD, median (range); ESR: erythrocyte sedimentation rate; CRP: C-reactive protein; TSH: thyroid stimulant hormone; fT4: free thyroxine; fT3: free triiodothyronine; Anti-TPO: anti thyroid peroxidase antibody; Anti-TG: anti thyroglobulin antibody. * 3 Patients using levothyroxine before SAT diagnosis were excluded from the evaluation of permanent hypothyroidism.

## DISCUSSION

Ultrasound assessment plays an important role in the diagnosis, differential diagnosis and monitoring of SAT patients. However, as the relationship between ultrasonographic findings and severity or prognosis of the disease is not known, therefore, ultrasonographic findings are not used in the treatment selection. The ultrasonographic findings of subacute thyroiditis are well established, and typical ultrasonographic findings include focal or multifocal hypoechoic areas with blurred borders and reduced vascularization in one or both thyroid lobes ( 9 ). In the current study, the rate of bilateral disease was found to be 62% and no difference was found in WBC and neutrophil count and ESR and CRP levels between bilateral and unilateral disease groups. The higher fT4 level in the bilateral disease group is associated with the extent of the destruction in this group. The rate of bilateral disease is around 70% in the literature and the only factor that has been associated with bilateral or unilateral disease involvement is HLA type ( 10 - 12 ). There are few studies in literature which have investigated the relationship between the extent of hypoechoic areas and clinical severity. In the current study, the relationship was investigated between bilateral and unilateral disease involvement and clinical findings due to intermittent changes in localization and shape of hypoechoic areas ( 13 - 15 ). From the results of the current study, it was seen that bilateral or unilateral involvement of the disease at the time of diagnosis did not constitute a risk factor for recurrent disease or permanent hypothyroidism. To best of our knowledge, there is only one research in the literature that has evaluated the relationship between unilobular/bilobular disease involvement and permanent hypothyroidism rate ( 16 ). Nishihara et al. showed that bilateral disease involvement might be an indicator for the development of permanent hypothyroidism after SAT, which is contrary to our result. However, the follow-up period in this study was half of the current study and the accepted time for development of permanent hypothyroidism was three months which was relatively short ( 16 ). Bennedbaek and Hegedüs showed that hypoechoic areas exceed 75% of thyroid volume in more than half of SAT patients, but the width of these areas was not related to thyroid function or disease recurrence ( 17 ). Omori and cols. reported that the extent of hypoechoic areas was associated with pain and ft4 level, but was not a risk factor for permanent hypothyroidism ( 18 ).

A reduction of approximately 50% in thyroid volume was detected between the time of diagnosis and after completion of treatment. As basal ultrasonography data were not available in this study, no differentiation could be made between oedema and permanent damage effect in respect of volume change. The initial thyroid volume had no effect on recurrence, whereas the permanent hypothyroidism rate was higher in patients with a lower initial thyroid volume. Bennednaek and Hegedüs also reported a 68% reduction in thyroid volume at the end of treatment in SAT patients and initial thyroid volume was not related with recurrence rate in that study ( 17 ). Initial thyroid volume was not detected as an independent risk factor for developing permanent hypothyroidism. As expected, the rate of thyroid antibody positive patients was significantly higher in patients with permanent hypothyroidism. In literature, only the short duration of treatment and HLA type have been associated with relapse and no risk factor for hypothyroidism has been identified ( 8 , 19 ). In the current study, anti-TPO positivity was detected as a risk factor for permanent hypothyroidism, whereas bilateral or unilateral disease involvement was not an indicator of recurrence or permanent hypothyroidism.

In conclusion, unlike anti-TPO positivity, ultrasonographic findings were not identified as a risk factor for recurrence and permanent hypothyroidism. The initial ultrasonographic appearance is not a determinant of choice of treatment. FT4 levels were found to be associated with the extension of the disease.
